# A role of the uterine cervix in the pathogenesis of adenomyosis and endometriosis?

**DOI:** 10.1002/ijgo.70287

**Published:** 2025-06-04

**Authors:** Angelo Cagnacci, Ambrogio Pietro Londero, Umberto Scovazzi, Anjeza Xholli

**Affiliations:** ^1^ Academic Unit of Obstetrics and Gynecology IRCCS San Martino Hospital Genoa Italy; ^2^ Department of Neurology, Rehabilitation, Ophthalmology, Genetics, Maternal, and Infant Health (DiNOGMI) IRCCS San Martino Hospital Genoa Italy; ^3^ Obstetrics and Gynecology Unit IRCCS Ospedale Pediatrico Giannina Gaslini Genoa Italy

**Keywords:** adenomyosis, cervix, dysmenorrhea, endometriosis, endometritis, infertility, pelvic inflammatory disease

## Abstract

Data indicate a link between the cervix and pathologies like adenomyosis and endometriosis. Uterine flexion on the cervix, diameter and stiffness of the internal cervical orifice represent an obstacle to downstream menstrual flow, that when excessive increases the intensity of uterine contractions. Evidence indicates that intense uterine contractions damage the endometrial myometrial junction, favoring endometrial entrance into the myometrium, to induce adenomyosis, and retrograde menstrual flow into the peritoneal cavity, to induce endometriosis. As a consequence of local hyperestrogenism in women with adenomyosis the cervical mucus barrier is reduced. Because of an increased prevalence of non‐lactobacilli dominated vagino‐types, the ascent of vaginal pathogens is also increased. Richness of endometrial and pelvic microbiota, lead to a higher rate of endometritis, and subclinical chronic pelvic infections in women with adenomyosis and concomitant endometriosis, respectively. Studies targeting the cervix as an actor on the pathogenesis of adenomyosis and endometriosis are warranted.

## 1 INTRODUCTION

The uterine cervix, which has long been recognized for its critical role as a barrier and support structure for fetal development, may have far‐reaching implications for women's reproductive health. Past and recent studies indicate that the cervix may be involved in the pathogenesis of gynecologic disorders like adenomyosis and endometriosis.

This article delves into the anatomic and functional structure of the cervix, to understand better how cervical anatomy and function variations may contribute to the development of these pathologies. The present study does not discuss in detail the different pathogenetic theories of adenomyosis and endometriosis, but rather outlines how the cervix can be involved in some pathogenetic mechanisms representing risk factors for these pathologies.

## 2 METHODS

A comprehensive literature search was conducted using PubMed and Scopus databases until October 2024. The search strategy used a combination of medical subject headings (MeSH) terms and free‐text keywords related to the cervix and the specific gynecologic pathologies. The following search terms were used separately and in various combinations: “cervix”, “cervical anatomy”, “internal cervical os”, “cervical canal”, “cervical stiffness”, “cervical elasticity”, “uterine flexion”, “uterine angle”, “dysmenorrhea”, “menstrual pain”, “adenomyosis”, “endometriosis”, “endometritis”, “pelvic inflammatory disease”, “infertility”, “preterm birth”, “menstrual flow”, “menstrual obstruction”, “retrograde menstruation”, “cervical mucus”, “mucus barrier”, “collagen fibers”, “muscle cells”, “innervation of cervix”, “estrogen production”, “oxidative stress”, “inflammation”, “microbiota” and “pathogenesis”.

The inclusion criteria included articles published in English that provided insights into the anatomy and physiology of the cervix, its role in menstrual flow, and its involvement in developing the aforementioned gynecologic conditions. We considered both original research articles and comprehensive reviews.

The initial search yielded a substantial number of articles whose titles and abstracts were evaluated for relevance. Full text versions of potentially relevant articles were retrieved and thoroughly reviewed. Additional references were found through manual searches of the bibliographies of selected papers or expert opinions. Thereafter, a selection of the most authoritative studies and reviews was performed to give a comprehensive overview of the current knowledge about the role of the cervix in the pathogenesis of adenomyosis and endometriosis.

## 3 STRUCTURE AND PHYSIOLOGY OF THE CERVIX

**FIGURE 1:**
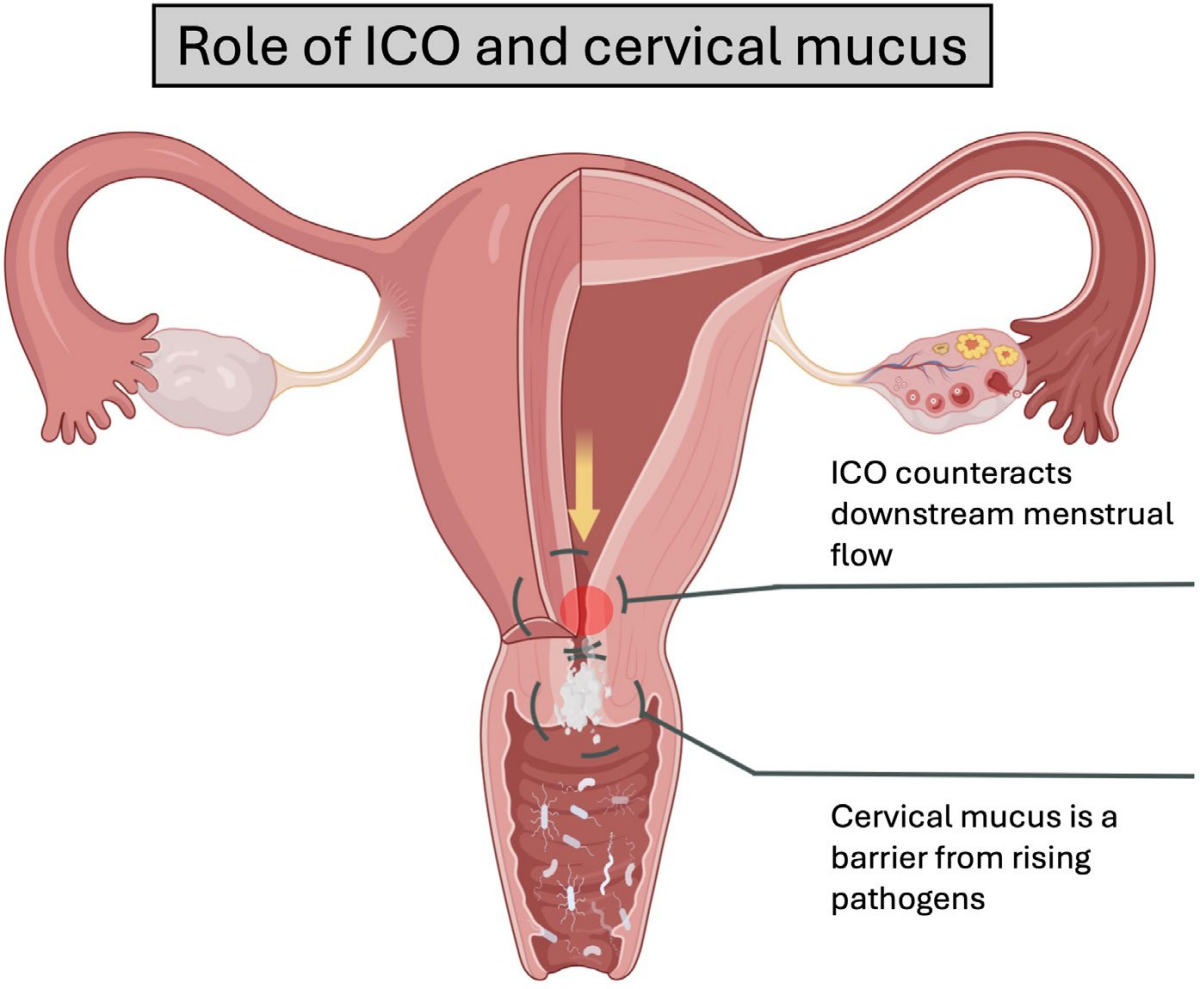
Physiological role of the internal cervical os (ICO) and cervical mucus.

The cervix is a fibromuscular cylindric structure constituted by a cervical canal surrounded by collagen and muscular tissue,^1^, ^2^ that is in a continuum with the corpus of the uterus. Its length is around 4 cm, and its diameter is about 2.5–3 cm, larger in parous than nulliparous women.^3^ The cervical canal through the internal cervical orifice (os), communicates with the endometrial cavity and through the external cervical os, with the vagina. It is lined by a glandular epithelium,^3^ that produces most of the cervical mucus. The cervical mucus is composed of water, mucins and dispersed immunoglobulins, immune cells, antimicrobial peptides and exfoliating epithelial cells.^4^ Estrogen stimuli increase mucous quantity and decrease its viscosity and the content of immune cells. Estrogens also increase the pore diameter of the mucin protein network favoring the access of spermatozoa to the internal genitalia. The opposite is exerted by progesterone. The barrier represented by the cervical mucus also opposes the ascent of bacteria from the vagina^4^ (Figure 1). This property is favored by the interaction of mucin with lactic acid and the low pH of lactobacilli‐dominated vagino‐types.^4^ The barrier is partially deranged in non‐lactobacilli‐dominated vagino‐types characterized by an unstable equilibrium among different bacteria (class IV vagino‐types).^4^

Cervical tissue around the canal, is formed by collagen fibers and muscular cells embedded in a collagen matrix.^1^, ^2^ The distribution and quantity of collagen fibers is influenced by forces applied to the cervix, and along with collagen matrix and muscle cells, determine a different degree of tissue stiffness in different areas.^5^ The muscular component of the cervix represents about 50%–60% of the tissue around the internal cervical os and at this level it is structured in an internal longitudinal layer close to the cervical canal and an external circumferential layer forming a presumed muscular orifice. Muscle cells decrease to 40% at the mid‐cervix level, conserving the same spatial distribution, and are very few (10%) and randomly scattered at the external cervical os.^2^ Muscle cells contract under oxytocin with an intensity that is double at the internal than external cervical os.^2^ Calcium antagonists counteract this effect.^2^ The tonic closure of the internal cervical os has a role of containment and represent a barrier to menstrual flow (Figure 1). At an ultrasound evaluation by strain elastography, it is the stiffest or less elastic structure of the cervix.^5^

Sensitive and autonomic neuronal fibers innervate the cervix from the hypogastric, splenic, and vagus nerves.^6^ Autonomic innervation influences cervix blood flow, mucus secretion production, and muscle contraction. Cervical stimulation induces sexual responses,^6^ and dilation of the internal cervical os, induces vasovagal responses,^7^ typical of women suffering from dysmenorrhea.

## 4 DYSMENORRHEA: POSSIBLE ROLE OF THE CERVIX

During menses uterine contractions aim to expel shed endometrium and blood through the cervix into the vagina. In this context, the cervix represents a downstream obstacle to menstrual flow. In a previous investigation it was found that uterine contractions are painful in about 85% (*n* = 343/408) of young women.^8^ Dysmenorrhea affected 25% of these women (*n* = 103/408) and was considered a condition in which menstrual pain is associated with vagal symptoms impeding normal daily activities and is so severe to require the use of painkillers.^8^ In 88% of cases (*n* = 359/408), dysmenorrhea started within the first year since menarche,^8^ in the absence of pathologies like endometriosis or adenomyosis. Dysmenorrhea can also be identified by the presence of menstrual pain with a value above 4, on a 10 cm visual analog scale (VAS). VAS values greater than 7 are considered severe dysmenorrhea.^8^, ^9^

Peculiar characteristics of the cervix may increase the downstream obstacle to menstrual flow. It was estimated that an angle of flexion of the uterus on the cervix greater than 210° (retroflexed uterus) is associated with the highest VAS values for menstrual pain. Intermediate VAS values were observed with an angle less than 150° (anti‐flexed uterus), and lower VAS values with an angle of flexion between 150° and 210° (linear uterus). Accordingly, severe dysmenorrhea (VAS > 7) was more prevalent with an angle of flexion greater than 210° (*n* = 17/22, 77.7%, *P* = 0.001) than with an angle less than 150° (*n* = 56/124, 45.2%) and an angle between 150° and 210° (*n* = 11/35, 31.4%).^9^ A downstream obstacle to menstrual flow can also be exerted by the internal cervical os and, to a lesser extent, by the length of the cervix.^10^ In 75 women, stiffness of the internal cervical os, measured by ultrasound strain elastography,^11^ was linearly associated with a progressive increase (*P* = 0.01) of menstrual pain, measured by VAS.

## 5 ADENOMYOSIS: POSSIBLE ROLE OF THE CERVIX

**FIGURE 2:**
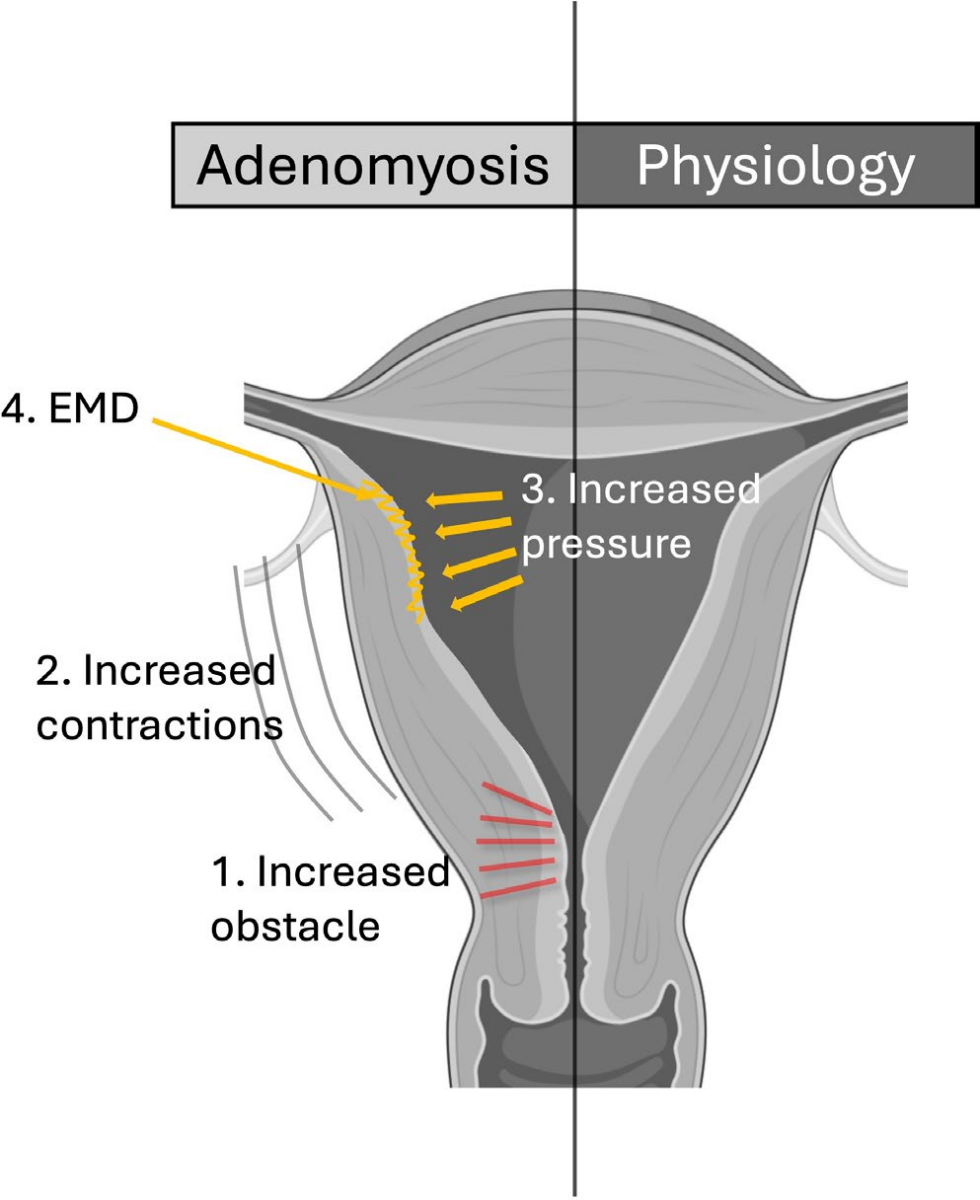
Schematic representation of the role of the cervix in the pathogenetic pathway of adenomyosis. EMD, endometrial‐myometrial interface disruption.

Intense myometrial contractions in women with dysmenorrhea increase the intraendometrial cavity pressure to values that may reach 300 mmHg.^12^ This increased pressure is exerted on the endometrial myometrial interface that, close to the fundus between the two ostial os, is stretched by divergent forces.^12^ The intraendometrial pressure and the strength of the stretching forces may contribute to the endometrial‐myometrial interface disruption (EMD) favoring the entrance of stem cells, epithelial cells, and stroma into the myometrium^12^ (Figure 2). In this mechanistic view, dysmenorrhea, a main symptom of adenomyosis, precedes and does not follow, the formation of adenomyotic lesions, and the risk factors for dysmenorrhea represent also the risk factors for adenomyosis.

Indeed, a higher prevalence of retroflexed uteri (angle >210°) that is a risk factor for severe dysmenorrhea, was observed in women with (19/76; 25%) than without (3/44; 6.8%) adenomyosis (*P* = 0.015),^13^ and the odds ratio of suffering from adenomyosis was 5.80 (95% confidence interval [CI]: 1.19/28.30; *P* = 0.029) in women with an angle of flexion greater than 210°. Similarly, stiffness of the internal cervical os, another risk factor for dysmenorrhea, was higher in 103 women with than in 172 women without adenomyosis and represented an independent risk factor of adenomyosis.^14^

**FIGURE 3:**
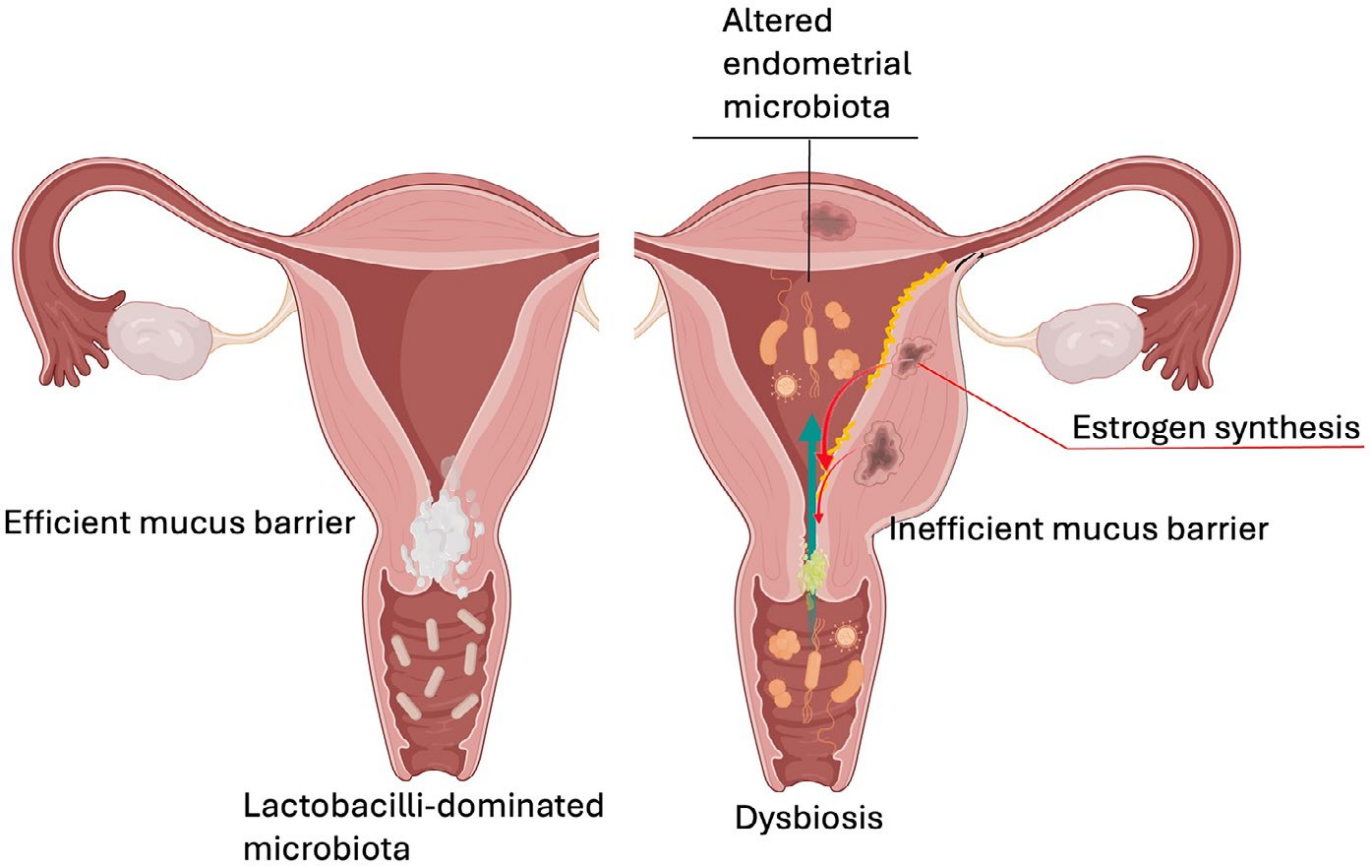
Mechanisms favoring an altered endometrial microbiota in women with adenomyosis. An increased production of estrogens by adenomyotic cells reduces the cervical mucus barrier with the ascent of bacterial pathogen from a vagina with dysbiosis.

Adenomyotic lesions are rich in aromatase and are capable to convert testosterone to estradiol.^15^ Accordingly, the menstrual content of estrogen of women with adenomyosis is higher.^15^ This local estrogen stimulus may have an influence on cervical mucus permeability (Figure 3). Elegant studies with radioactive tracer introduced into the vagina have clearly shown that in the early follicular phase of women suffering from adenomyosis, the tracer enters the endometrial cavity, while in control women, it remains confined to the vagina.^16^ Similarly, in the mid‐follicular phase, in women with adenomyosis the radioactive tracer massively enters the endometrial cavity and pelvis, while in control women it only minimally enters the endometrial cavity. Women with adenomyosis have a higher prevalence of non‐lactobacilli dominated vagino‐types^17^ and because they have a higher cervical mucus permeability, they show the same bacteria prevalence in the endometrium (Figure 3). As a consequence, the prevalence of chronic endometritis is higher in women with adenomyosis,^18^ contributing to a higher rate of infertility, fallopian tube closure, and during pregnancy to a higher rate of miscarriages, intrauterine infections and preterm birth.^19^, ^20^ Thus, the clinical manifestation and consequences of adenomyosis may still recognize a pathogenetic role in the alteration of the cervical mucus barrier.

## 6 ENDOMETRIOSIS: POSSIBLE ROLE OF THE CERVIX

During the years several theories have been formulated on the pathogenesis of endometriosis. Without entering into the details of the different theories, it can be considered that the retrograde menstruation hypothesis can be applied to most of the endometriosis cases and can be part of the different pathogenetic theories. Via retrograde menstruation, endometrial but also stem cells contained in the menstrual flow enter the abdominal cavity and may implant in the different structures of the pelvis.^21^ Menstrual blood by increasing local oxidative stress may also represent a stimulus to the differentiation of peritoneal stem cells.^22^ A total of 90% of menstruating women experience retrograde menstruation, but not all develop endometriosis.^23^ The presence of concomitant facilitating factors, like local immune function derangement, increased endometrial cells adhesiveness, alteration of molecules involved in attachment and invasion, local hormone and cytokines production, and inducible local enzymes, are considered critical factors for its development.^21^ Yet, many of these factors can be activated as a consequence of the elevated quantity of iron brought by an excessive retrograde menstruation. In that condition scavenging defenses of the peritoneum can be overloaded favoring the adhesion of stem, endometrial and mesothelial cells, or activating local stem cells.^21^, ^22^ It cannot be excluded that in women with predisposition this pathogenetic mechanism is activated by a limited amount of blood, but it is likely that endometriosis is favored by higher quantities of blood consequent to excessive retrograde menstruation. The consequent cytokine production, immune local immune modifications, and neovascularization contribute to disease progression.^21^, ^22^

**FIGURE 4:**
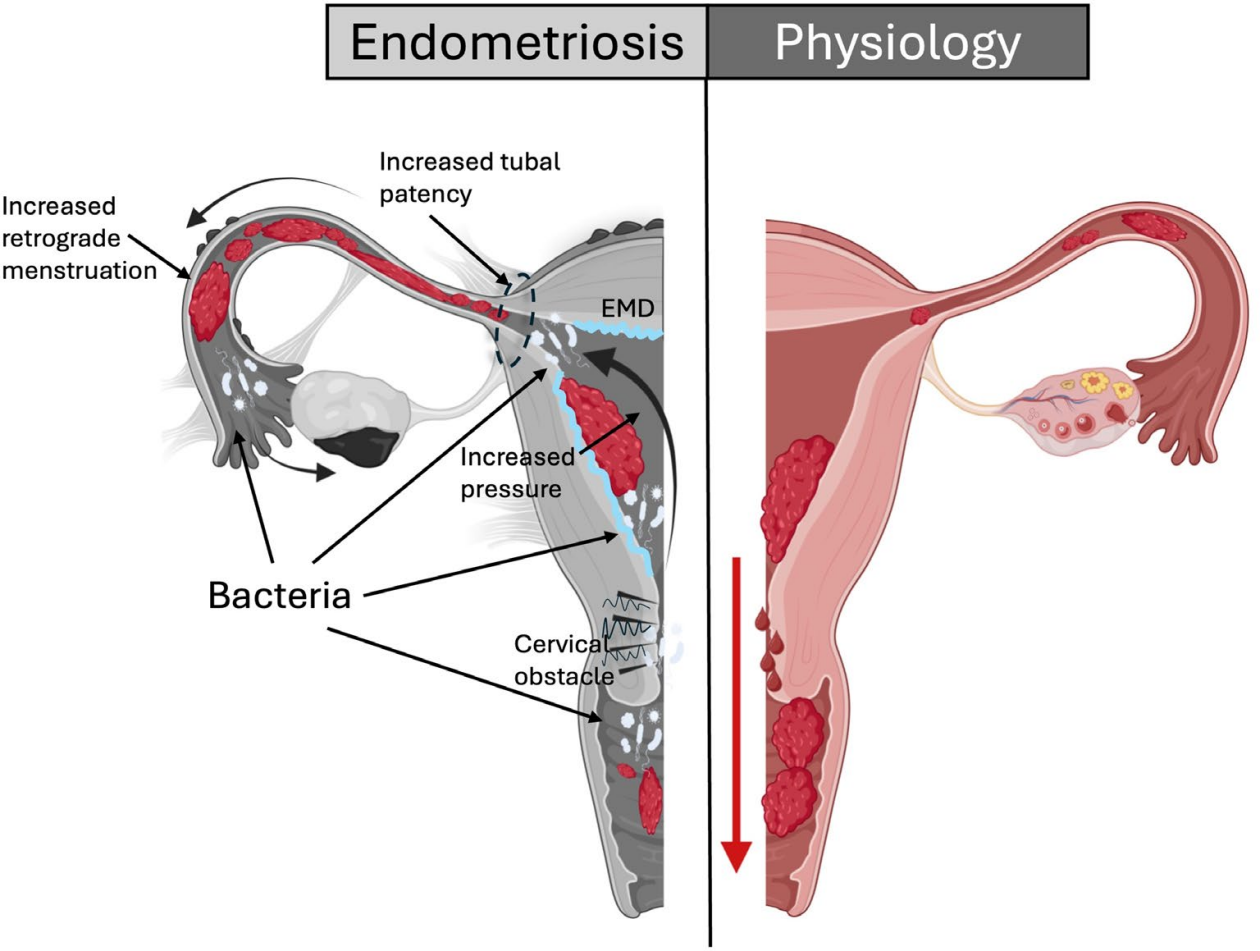
Schematic representation showing how an increased cervical obstacle can lead to endometriosis.

In this view, retrograde menstruation, particularly when it is excessive, represents a risk factor for pelvic endometriosis. Indeed, risk factors for endometriosis are conditions leading to an excessive exposure to retrograde menstruation as an earlier menarche, nulliparity, shorter intervals between cycles and prolonged menstruation.^21^, ^24^ Endometriosis is more frequent in women with uterine anatomic conformations favoring retrograde menstruation like tube hypotonia.^25^ Similarly, the interstitial portion of the tube is more frequently straight than curvilinear in women with (26/45; 57.8%) than without (25/182; 13.8%) endometriosis^26^ (Figure 4).

Elegant mathematical models have also indicated that the downstream obstacle represented by the cervix influence the directionality of menstrual flow and consequently the amount of retrograde menstruation^10^ (Figure 4). At an estimated intracavitary pressure of 80 mmHg, with a 10 mm cervix length, and fixed characteristics of the tubes, the flow within the experimental tubes increases exponentially with a diameter of the internal cervical os <3 mm. In cases of longer cervix (length of 50 mm), this exponential increase of flow in the experimental tubes starts with an internal cervical os diameter of 4 mm. In baboons, supracervical ligation induces the development of endometriosis,^27^ and endometriosis was found in women with obstructive congenital Mullerian abnormalities.^28^ In infertile women with cervical stenosis the rate of endometriosis (11/84; 13.1%) is higher than in infertile women without (3/84; 2.6%) cervical stenosis (*P* = 0.005).^29^ Similarly, cervical stenosis, defined in surgical specimens as an internal cervical os diameter less than 4.5 mm, was associated with the presence of pelvic endometriosis in 24/25 cases.^30^ In our hands, in a study performed on 287 women of which 157 with and 130 without endometriosis, stiffness of the internal cervical os was associated with a greater risk of endometriosis.^31^ The association remained even when women with concomitant adenomyosis were excluded. In the same study, the degree of the angle of uterine flexion was also an independent determinant of the risk of endometriosis (OR for each degree 1.01, 95% CI: 1.00/1.01; *P* = 0.034).

Alterations in the gut, vaginal and endometrial microbiota were reported in women with endometriosis.^32^, ^33^, ^34^, ^35^ Different mechanisms have been advocated to explain the link between gut microbiota alteration and endometriosis, and the directionality of the association is not completely understood.^32^ Gut may influence vaginal microbiota and when endometriosis is concomitant to adenomyosis the opening of the cervical mucous gate, consequent to local hyperestrogenic conditions induced by adenomyotic foci, may favor the entrance of pathogens that can be easily transported in the peritoneal cavity (Figure 4). Richness of peritoneal bacteria has been described in women with endometriosis, with similarities to those found in the vaginal fluid^32^ and this may contribute to pelvic inflammation, clinical symptoms and disease progression.^35^

## 7 FUTURE PROSPECTIVES

In the present study we concentrated on literature dealing with the role possibly played by the cervix on the pathogenesis of adenomyosis and endometriosis without addressing other proposed pathogenetic mechanisms. The present study was based on condensing different types of data, mostly observational and experimental. Elastography data may have weaknesses due to reproducibility, but the cited articles all used methods to make the analysis reliable. None of the data by itself can sustain pathogenetic mechanisms, but their condensation seems to sustain a potential role of the cervix in the pathogenesis of adenomyosis and endometriosis. The cervix can play a dual pathogenetic role. An excessive obstacle exerted to downstream menstrual flow, leading to intense uterine contractions, dysmenorrhea and spontaneous disruption of the endometrial‐myometrial interface, in the case of adenomyosis, or increased retrograde menstruation, in the case of endometriosis, or both. As a consequence of local hyperestrogenism due to adenomyosis, the cervix loses its barrier capability, favoring the ascent of vaginal pathogens. Subclinical endometritis and pelvic infections and their consequences contribute to the development and clinical consequences of the diseases.

These data add scientific support to most of the therapeutical remedies used to treat and prevent adenomyosis and endometriosis. Both intense uterine contractions, favoring adenomyosis, and the extent of retrograde menstruation, to induce endometriosis, benefit from the use of hormonal therapies aimed to reduce or abolish menstrual flow.^36^ However, the benefits encompass the control of menstruation and impinge upon the modifications of the cervical mucus that progestin or estro‐progestin administration make less permeable to the ascent of vaginal pathogens.

To date, hormonal therapies represent the main therapeutical approach but in future they could be complemented by alternative remedies.

The obstacle to downstream menstrual flow may be further investigated to define how it can predict the risk of the disease, and studies aimed at determining how this obstacle can be decreased could be of interest. Some cervix characteristics, like the angle of uterine flexion, are probably less practical to correct, but methods alternative to surgery^37^ should be investigated. A promising approach could also be the modulation of the internal cervical os stiffness. A general or locally administered tool that modifies collagen composition or tonic contraction of muscle at the internal cervical os could possibly help to reduce the obstacle to menstrual flow. More studies should also concentrate on how to reduce vaginal dysbiosis of women with adenomyosis and endometriosis and modify the characteristic of the cervical mucus to make it less penetrable to vaginal pathogens.

Ultimately, investigations on the cervix and its possible modifications may lead to the development of a new therapeutic tool to complement or substitute those already present in our armamentarium for the prevention and treatment of these complex pathologies.

## AUTHOR CONTRIBUTIONS

A.C, A.P.L., U.S. and AX. conceptualization, draft preparation, writing, review editing, and approval of the final manuscript.

## FUNDING INFORMATION

This manuscript received no external funding.

## CONFLICT OF INTEREST STATEMENT

None of the authors declare any conflict of interest.

## Data Availability

Data sharing is not applicable to this article as no new data were created or analyzed in this study.
